# Automated deep learning for classification of dental implant radiographs using a large multi-center dataset

**DOI:** 10.1038/s41598-023-32118-1

**Published:** 2023-03-24

**Authors:** Wonse Park, Jong-Ki Huh, Jae-Hong Lee

**Affiliations:** 1 Korean Academy of Oral and Maxillofacial Implantology (KAOMI) Implant Research Institute, Seoul, Korea; 2 Department of Advanced General Dentistry, Yonsei University College of Dentistry, Seoul, Korea; 3 Department of Oral and Maxillofacial Surgery, Gangnam Severance Hospital, Yonsei University College of Dentistry, 211 Eonju-ro, Gangnam-gu, Seoul, 06273 Korea; 4 Department of Periodontology, College of Dentistry and Institute of Oral Bioscience, Jeonbuk National University, 567 Baekje-daero, Deokjin-gu, Jeonju, 54896 Korea

**Keywords:** Dental materials, Dental radiology

## Abstract

This study aimed to evaluate the accuracy of automated deep learning (DL) algorithm for identifying and classifying various types of dental implant systems (DIS) using a large-scale multicenter dataset. Dental implant radiographs of pos-implant surgery were collected from five college dental hospitals and 10 private dental clinics, and validated by the National Information Society Agency and the Korean Academy of Oral and Maxillofacial Implantology. The dataset contained a total of 156,965 panoramic and periapical radiographic images and comprised 10 manufacturers and 27 different types of DIS. The accuracy, precision, recall, F1 score, and confusion matrix were calculated to evaluate the classification performance of the automated DL algorithm. The performance metrics of the automated DL based on accuracy, precision, recall, and F1 score for 116,756 panoramic and 40,209 periapical radiographic images were 88.53%, 85.70%, 82.30%, and 84.00%, respectively. Using only panoramic images, the DL algorithm achieved 87.89% accuracy, 85.20% precision, 81.10% recall, and 83.10% F1 score, whereas the corresponding values using only periapical images achieved 86.87% accuracy, 84.40% precision, 81.70% recall, and 83.00% F1 score, respectively. Within the study limitations, automated DL shows a reliable classification accuracy based on large-scale and comprehensive datasets. Moreover, we observed no statistically significant difference in accuracy performance between the panoramic and periapical images. The clinical feasibility of the automated DL algorithm requires further confirmation using additional clinical datasets.

Dental implants are among the most widely used and commonly accepted treatment modalities for oral rehabilitation of partially and completely edentulous patients^1,2^. The occurrence of various major or critical mechanical (such as fractures of screws or fixtures) and biological (such as peri-implantitis) problems is steadily and inevitably increasing, affecting long-term survival and reintervention outcomes^3,4^. Therefore, dental implant-related complications are a growing concern in the dental community worldwide and are a public health problem associated with a high socio-economic burden^5,6^.

In particular, early detection and appropriate treatment of simple mechanical complications such as screw loosening can prevent more severe complications, such as fixture fracture or severe peri-implantitis, at an early stage^7,8^. For early and fast intervention, dental implant systems (DIS) placed in the oral cavity must be unambiguously identified and classified. However, in actual clinical practice, it is not easy to properly identify or classify the various different types of DIS after implant surgery because of various clinical and environmental factors, including the closure of a dental hospital or the loss of dental records.

Although two-dimensional dental radiography, including panoramic and periapical radiographs, is the most useful tool for identifying and classifying DIS post-implant surgery, there is a fundamental and practical limit for classifying thousands of different types of DIS with similar shapes and physical properties^9,10^. In addition, three-dimensional cone-beam computed tomography (CBCT) has been actively used for dental implant surgery; however, whether CBCT can better classify DIS is debatable because the sharpness and resolution of CBCT is still significantly lower than that of peripheral radiographs^11^.

Deep learning (DL), a subfield of artificial intelligence (AI), has a wide range of applications in medicine; this unique technology is associated with high accuracy in medical image analysis for edge detection, classification, or segmentation based on a cascade of multiple computational and hidden layers in a deep neural network^12^. When limited to dentistry, deep and convolutional neural networks have rapidly become the methodology of choice for two- and three-dimensional dental image analyses^13–16^. Several studies have demonstrated DL algorithms as an emerging state-of-the-art approach in terms of accuracy performance for identifying and classifying various types of DIS and often show outperforming results compared to dental professionals specialized in implantology^17–25^. However, since most previous studies were based on fewer than thousands of DIS images or fewer than 10 different types of DIS, available evidence is insufficient to be implemented in actual clinical practice^19–24^. This study aimed to evaluate the accuracy of the automated DL algorithm for the identification and classification of DIS using a large-scale and comprehensive multicenter dataset.

## Results

The performance metrics of the automated DL algorithm based on the accuracy, precision, recall, and F1 score for total of 156,965 panoramic and periapical radiographic images were 88.53%, 85.70%, 82.30%, and 84.00%, respectively. Using only panoramic images (*n* = 116,756), the DL algorithm achieved 87.89% accuracy, 85.20% precision, 81.10% recall, and 83.10% F1 score, whereas the corresponding values using only periapical images (*n* = 40,209) achieved 86.87% accuracy, 84.40% precision, 81.70% recall, and 83.00% F1 score, respectively. No statistically significant difference in the classification accuracy was observed between the three groups, and the detailed accuracy performances of DL in the classification of DIS are listed in Table 1.

**Table 1:** Accuracy performance of automated deep learning algorithm.

Manufactures	System	Panoramic images (Accuracy = 87.89%)			Periapical images (Accuracy = 86.87%)			Panoramic and Periapical images (Accuracy = 88.53%)		
		Precision (%)	Recall (%)	F1 score (%)	Precision (%)	Recall (%)	F1 score (%)	Precision (%)	Recall (%)	F1 score (%)
All dental implant systems		85.20	81.10	83.10	84.40	81.70	83.00	85.70	82.30	84.00
Neobiotech	IS I	88.30	93.00	90.60	87.60	93.00	90.20	91.30	92.10	91.70
	IS II	66.70	20.00	30.80	60.00	30.00	40.00	66.70	20.00	30.80
	IS III	87.80	81.10	84.30	74.10	75.50	74.80	79.40	94.30	86.20
	EB	94.00	90.40	92.20	98.00	92.30	95.00	97.90	88.50	92.90
Nobel biocare	Branemark	92.90	76.50	83.90	96.00	70.60	81.40	100.0	76.50	86.70
Dentsply	Astra	93.40	100.0	96.60	93.40	100.0	96.60	93.40	100.0	96.60
	Xive	98.50	100.0	99.20	100.0	100.0	100.0	98.50	100.0	99.20
Dentium	Implantium	95.20	96.20	95.70	95.90	95.20	95.50	95.40	95.70	95.60
	Superline	95.00	95.40	95.20	94.20	93.70	93.90	96.00	95.60	95.80
Dioimplant	UF	92.90	96.30	94.50	96.20	92.60	94.30	100.0	96.30	98.10
	UF II	86.20	86.20	86.20	86.20	86.20	86.20	86.20	86.20	86.20
Megagen	Any ridge	92.30	92.30	92.30	85.70	92.30	88.90	84.60	84.60	84.60
	Anyone internal	73.80	82.90	78.10	72.0	71.90	72.40	79.40	64.10	70.90
	Anyone external	66.40	59.50	62.80	58.00	65.909	61.70	56.50	76.20	64.90
	Exfeel external	100.0	75.0	85.70	100.0	83.30	90.90	100.0	83.30	90.90
Straumann	TS standard	84.20	88.90	86.50	88.90	88.90	88.90	84.20	88.90	86.50
	TS standard plus	90.30	93.30	91.80	93.80	100.0	96.80	93.50	96.70	95.10
	Bone level	99.20	96.00	97.60	98.40	96.00	97.20	99.20	94.40	96.70
Shinhung	Luna	97.50	88.60	92.90	83.00	88.60	85.70	92.90	88.60	90.70
Osstem	GS II	86.10	93.90	89.90	93.80	90.90	92.30	81.60	93.90	87.30
	SS II	77.80	58.30	66.70	90.00	75.00	81.80	80.00	66.70	72.70
	TS III	97.40	96.80	97.10	97.30	96.20	96.80	97.60	97.60	97.60
	US II	91.50	95.30	93.40	90.50	97.00	93.70	91.00	98.70	94.70
	US III	100.00	78.90	88.20	93.30	73.70	82.40	93.30	73.70	82.40
Warantec	Hexplant	80.20	64.60	71.60	79.60	62.10	69.70	78.90	72.80	75.80
	Internal	54.50	71.50	61.80	52.50	71.90	61.30	60.10	67.20	63.50
	IT	19.00	19.00	19.00	19.20	23.80	21.30	35.30	28.60	31.60

**Figure 1:**
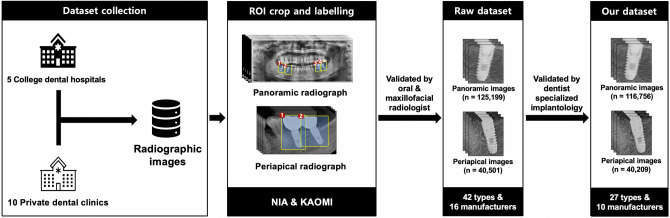
Schematic illustration of dataset collection and verification. All study protocols and related procedures were supervised by the National Information Society Agency (NIA) and the Korean Academy of Oral & Maxillofacial Implantology (KAOMI).

Figure 1 shows the normalized confusion matrices, containing a summary of the classification of the 27 different types of DIS based on the automated DL algorithm (full details are provided in Appendix 3). Using panoramic and periapical images, the classification accuracy of DL was the highest for Nobel Biocare Branemark (100.0%) and Megagen Exfeel external (100.0%), and the lowest for Warantec IT (35.3%). Using only panoramic images, the classification accuracy was the highest for Osstem US III (100.0%) and Megagen Exfeel external (100.0%), and the lowest for Warantec IT (19.0%). When using only periapical images, the classification accuracy was the highest for Megagen Exfeel external (100.0%) and Dentsply Xive (100.0%), and the lowest for Warantec IT (19.2%).

## Discussion

AI-based large-scale machine learning and DL in the late 2010s, which facilitated the accurate diagnosis of medical radiographic images, garnered attention in biomedical engineering and provided novel insights into precision medicine^26–28^. More recently, deep convolutional neural network algorithms have gained popularity in dentistry, and have also achieved considerable success in analyzing dental radiographic images^29^. The potential clinical applications of DL technology are closely related to (1) deeper and more sophisticated neural network structures and (2) large annotated and high-quality datasets. Particularly, a gold-standard dataset annotated and verified by medical and dental professionals is essential to create a reliable radiographic image-based DL model in the medical and dental fields^26,27^.

To evaluate the performance of DL-based identification and classification of various types of DIS in actual clinical practice, a large, highly accurate, and reliable dataset is necessary. Recently, a large-scale and comprehensive multicenter dataset that could be used in the clinical field for DL-based identification and classification of DIS was collected and released openly by the national initiative. To our knowledge, the dataset used in the present study contained the larger number of radiographic images and types of DIS than any previously reported implant-related dataset. Because we used this dataset in the current study, it is expected to show higher feasibility than that of any previous implant-related DL research.

Most previous studies evaluated the accuracy performance of the conventional or minimally modified DL architectures (e.g., YOLO, SqueezeNet, ResNet, GoogLeNet, and VGG-16/19) using less than a few thousand dental radiographic images, and usually fewer than 10 different types of DIS in their datasets, identifying a classification accuracy ranging from 70 to 100%^17–25^. One study that utilized a ResNet architecture based on 12 types of 9767 panoramic images reported a high accuracy of 98% or more^23^. Our previous pilot study that utilized automated DL based on six different types of 11,980 DIS images also showed reliable outcomes and achieved a very high accuracy of 95.4% (sensitivity:95.5% and specificity:85.3%)^18^. Conversely, another study based on Yolov3 using 1282 panoramic images showed a relatively low accuracy in the 70% range on average^22^.

The automated DL algorithm used in this study, based on the combination of periapical and panoramic radiographs, achieved an AUC of 0.885. When only panoramic radiographs were used, the AUC was 0.878, and when only periapical radiographs were used, the AUC was 0.868. Specifically, periapical and panoramic images had the highest classification accuracy, and periapical images alone had the lowest accuracy, but there was no statistically significant difference between the three groups. These outcomes are consistent with the previously reported absence of a significant difference in classification accuracy between panoramic and periapical images and are also likely due to the fact that almost three times more panoramic images (*n* = 105,080) than periapical images (*n* = 36,188) were used for training and validation^17,18^.

Specifically, the Nobel Biocare Branemark, Megagen Exfeel external, Osstem US III, and Dentsply Xive showed a high classification accuracy of 100.0%, whereas Warantec IT showed a low accuracy performance (accuracy: 19.0–35.3%) due to the relatively small number of radiographic images, including only 238 panoramic and 208 periapical images, despite having a conventional fixture morphology with an internally tapered shape. From this perspective, DL has great advantages in identifying and classifying similar types of DIS; however, the accuracy performance varies significantly depending on the amount of datasets required for training, which is considered a fundamental limitation of the existing DL algorithms. Further research should be conducted to confirm whether the number of datasets required for training can be reduced by adopting an algorithm that is more specialized than the algorithm in this study for DIS classification.

In the radiographs used in this study, the main ROI was the implant fixture, but a number of other confounding conditions (such as surrounding alveolar bone, cover screw, healing abutment, provisional or definitive prosthesis) were included. To be used in actual clinical practice, implant fixtures with different confounding conditions and angles should be used as datasets, rather than implant fixtures with perfect/intact shapes and standard angles. Several previous studies, including this one, have confirmed that implant datasets with different angles and confounding conditions have a high accuracy performance of over 80%^17,18,25^. Furthermore, using the Gradient-Weighted Class Activation Mapping technique, it was found that the types of DIS were classified by focusing on the implant fixture itself rather than the various confounding components of the DIS. Therefore, various confounding factors and angles do not appear to have a significant impact on the accuracy performance of DL-based implant system classification.

In a recent study wherein healthcare professionals with no coding experience evaluated the feasibility of automated DL models using five publicly available and open-source medical image datasets, most classification models showed accuracy performance and diagnostic properties comparable to those of state-of-the-art DL algorithms^30^. Developing customized DL models according to the types and characteristics of datasets requires highly specialized skills and expertise. This study confirmed that the DL algorithm itself, not computer scientists and engineers, built an automated DL model without coding and showed excellent classification accuracy of over 86% in 27 similar design but different types of multiple classifications.

Identifying and classifying DIS with varying features and characteristics and limited clinical and radiographic information is a challenge not only for inexperienced dental professionals, but also for dentists with sufficient experience in implant surgery and prosthetics. In the past, several studies have identified DIS from a forensic perspective based on radiographs, and until recently, efforts have been made to classify DIS, but most of these are based on empirical evidence, making it difficult to achieve high reliability^9,10,31^. More recently, computer-based implant recognition software and web-based DIS classification platforms have been developed and used; however, most require manual classification of DIS features (such as coronal interface, flange, thread type, taper and apex shape) or contain only a small number of DIS datasets, limiting their active use in clinical practice^32^.

The first end goal based on this research was to obtain a database of almost all types of DIS used worldwide and train it with sophisticated and refined DL algorithms optimized for DIS classification to achieve a high level of reliability that can be used in actual clinical practice. The second goal was to create a web or cloud-based environment where datasets can be freely stored, trained, and validated in real time. Achieving these goals requires the proactive development of standard protocols to facilitate data sharing and integration, secure transmission and storage of large datasets, and enable federated learning^33,34^.

This study had several limitations. Collecting a dataset using supervised learning requires considerable tangible and intangible resources including finances, time, trained personnel, hardware, and software. Therefore, unsupervised learning, a technique for overcoming small-scale and imbalanced datasets, has been introduced and tested with caution in dentistry; however, it remains a challenging approach^35^. Large-scale and multicenter datasets may be useful for future DL-based research and actual clinical trials to identify and classify various types of DIS. Nevertheless, the dataset used in this study had inherent limitations regarding the interpretability of the results. Although the raw NIA dataset consisted of 165,700 radiographs and 42 different types of DIS, the number of panoramic and periapical images for each type of DIS was highly heterogeneous. In addition, DIS manufactured by foreign companies or using non-titanium materials (such as non-metallic ceramic zirconia), which are rarely used in South Korea, were few or not included in the raw dataset. To overcome the potential problem of overfitting and selective bias, we selected only DIS that contained more than 100 images of panoramic and periapical radiographs.

## Conclusion

We verified that automated DL shows a high classification accuracy based on large-scale and multicenter datasets. Furthermore, no significant difference in accuracy was observed between panoramic and periapical radiographic images. The clinical feasibility of the automated DL algorithm will have to be confirmed using additional datasets and clinical research in the future.

## Materials and methods

### Ethics

This study was approved and conducted in accordance with the following Institutional Review Board (IRB): Seoul National University Dental Hospital (ERI21024), Yonsei University Dental Hospital (2-2021-0049), Gangnam Severance Dental Hospital (3-2021-0175), Wonkwang University Daejeon Dental Hospital (W2104/003-002), Dankook University Dental Hospital (2021-8-004), and national public IRB (P01-202109-21-020). IRBs of Seoul National University Dental Hospital, Yonsei University Dental Hospital, Gangnam Severance Dental Hospital, Wonkwang University Daejeon Dental Hospital, Dankook University Dental Hospital, and national public approved a waiver of informed consent for retrospective large-scale and multicenter data analysis. All methods in this study were performed in accordance to relative guidelines and regulations.

### Dataset collection and verification

All included dental radiographic images were managed and supervised by the National Information Society Agency (NIA) under the Ministry of Science and the Korean Academy of Oral and Maxillofacial Implantology (KAOMI). The dataset was collected from five college dental hospitals (including Seoul National University Dental Hospital, Yonsei University Dental Hospital, Yonsei University Gangnam Severance Dental Hospital, Wonkwang University Daejeon Dental Hospital, and Dankook University Dental Hospital) and 10 private dental clinics. Appendix 1 summarizes the detailed consortium of the dataset collection.

**Figure 2:**
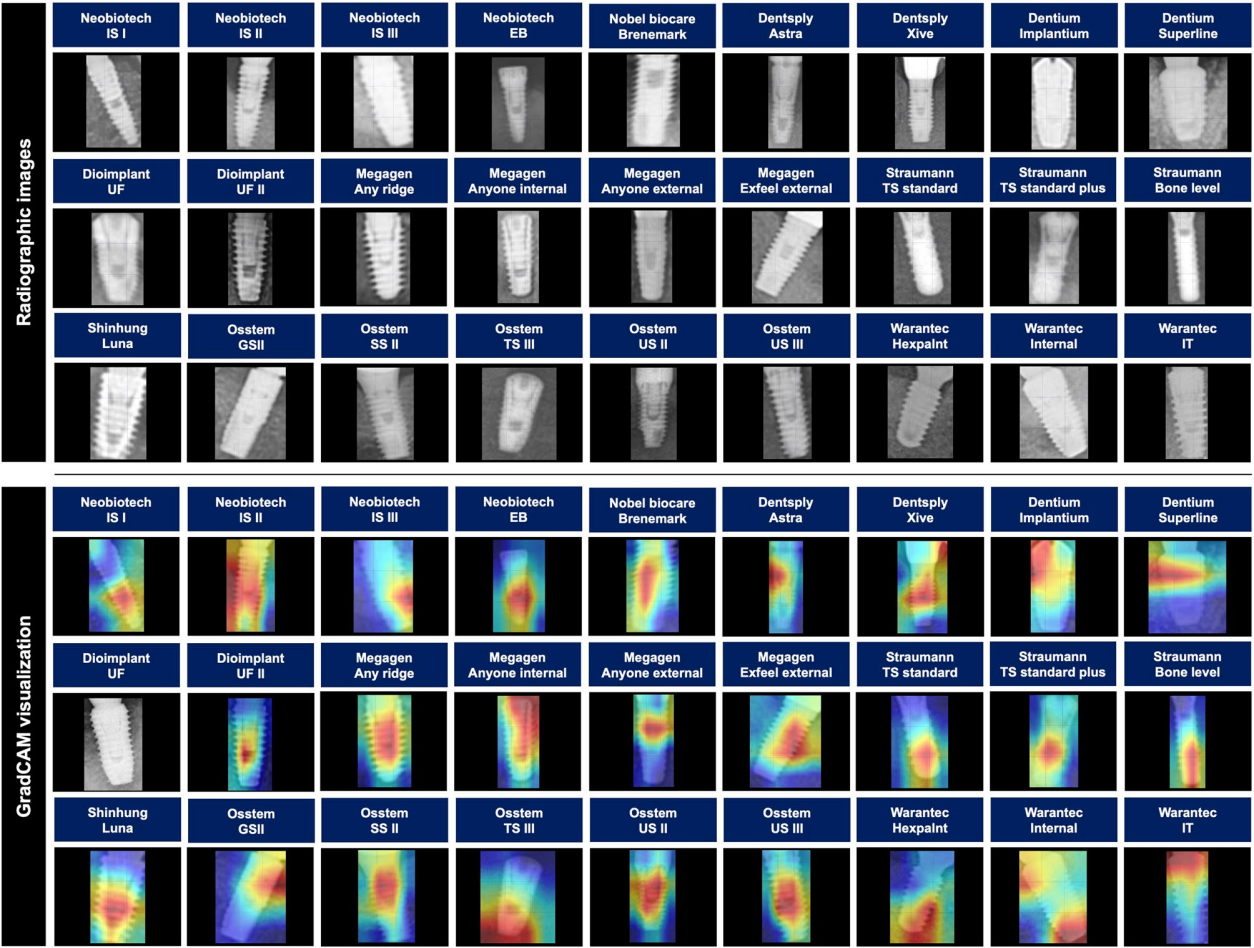
Dataset containing 116,756 panoramic and 40,209 periapical radiographic images and comprising 10 manufacturers and 27 types of dental implant systems.

Digital imaging and communication in medicine-format panoramic and periapical images were converted into either de-identified 512 × 512 pixels or smaller JPEG- or PNG-format images, and one implant fixture per image was cropped as a region of interest (ROI). The collected ROI images were reviewed to ensure that cropping, resolution, and sharpness were properly performed by 10 dental professionals employed by the KAOMI. Subsequently, based on the medical and dental records provided by college dental hospitals and private clinics, each implant fixture was labeled with the manufacturer, brand and system, diameter and length, placement position, surgery date, age, and sex using customized labeling and annotation tools. All included radiographic images were validated by a board-certified oral and maxillofacial radiologist who was not involved in dataset management. The final dataset consisted of 165,700 panoramic and periapical radiographic images and 42 types of DIS. Appendix 2 provides a detailed list of the raw NIA dataset (Fig. 2).

**Figure 3:**
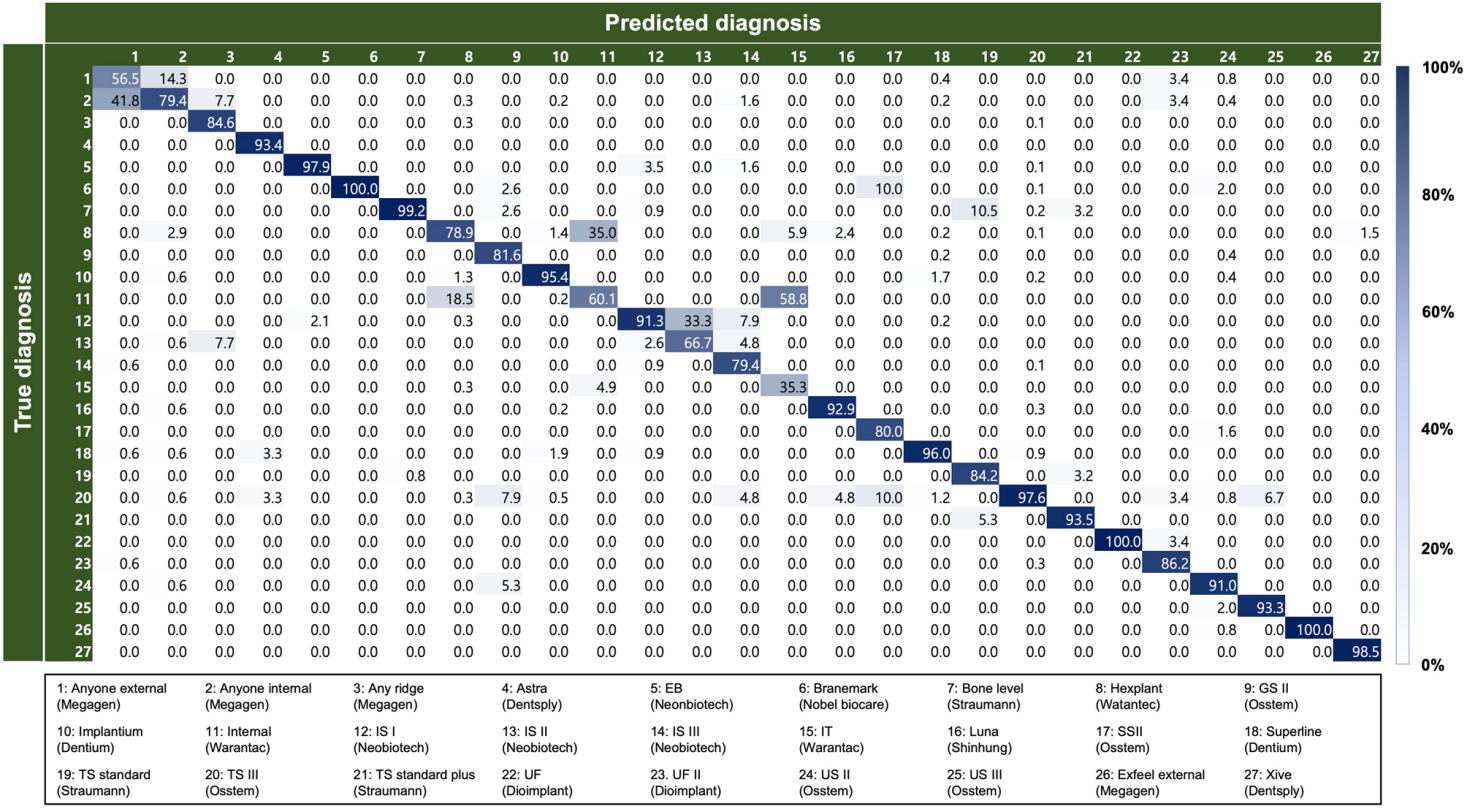
Multi-label classification confusion matrix with normalization using panoramic and periapical radiographic images.

We included only DIS that contained at least 100 periapical and 100 panoramic images from the raw NIA dataset. Finally, the dataset used in this study contained 116,756 panoramic and 40,209 periapical images, comprised 10 manufacturers and 27 types of DIS. Specifically, the dataset included Neobiotech (*n* = 21,260, 13.54%), Nobel biocare (*n* = 3644, 2.32%), Dentsply (*n* = 15,296, 9.74%), Dentium (*n* = 41,096, 26.18%), Dioimplant (*n* = 1530, 0.97%), Megagen (*n* = 7801, 4.97%), Straumann (*n* = 4977, 3.17%), Shinhung (*n* = 3376, 2.15%), Osstem (*n* = 42,920, 27.34%), and Warantec (*n* = 15,065, 9.60%). The detailed types of DIS are listed in Table 2 and illustrated in Fig. 3.

**Table 2:** Number of panoramic and periapical radiographic images for each dental implant system.

Manufactures	System	Panoramic images (*n* = 116,756)		Periapical images (*n* = 40,209)		Total images (*n* = 156,965)	
Neobiotech	IS I	6708	5.75%	1139	2.83%	7847	5.00%
	IS II	2774	2.38%	104	0.26%	2878	1.83%
	IS III	7594	6.50%	533	1.33%	8127	5.18%
	EB	1890	1.62%	518	1.29%	2408	1.53%
Nobel biocare	Branemark	3302	2.83%	342	0.85%	3644	2.32%
Dentsply	Astra	13,404	11.48%	571	1.42%	13,975	8.90%
	Xive	667	0.57%	654	1.63%	1321	0.84%
Dentium	Implantium	14,993	12.84%	4162	10.35%	19,155	12.20%
	Superline	16,734	14.33%	5207	12.95%	21,941	13.98%
Dioimplant	UF	525	0.45%	273	0.68%	798	0.51%
	UF II	447	0.38%	285	0.71%	732	0.47%
Megagen	Any ridge	217	0.19%	135	0.34%	352	0.22%
	Anyone internal	1640	1.40%	2167	5.39%	3807	2.43%
	Anyone external	1290	1.10%	1263	3.14%	2553	1.63%
	Exfeel external	974	0.83%	115	0.29%	1089	0.69%
Straumann	TS standard	1151	0.99%	176	0.44%	1327	0.85%
	TS standard plus	741	0.63%	301	0.75%	1042	0.66%
	Bone level	1347	1.15%	1261	3.14%	2608	1.66%
Shinhung	Luna	2935	2.51%	441	1.10%	3376	2.15%
Osstem	GS II	1401	1.20%	327	0.81%	1728	1.10%
	SS II	717	0.61%	116	0.29%	833	0.53%
	TS III	19,222	16.46%	10,536	26.20%	29,758	18.96%
	US II	7295	6.25%	2363	5.88%	9658	6.15%
	US III	758	0.65%	185	0.46%	943	0.60%
Warantec	Hexplant	4568	3.91%	4271	10.62%	8839	5.63%
	Internal	3224	2.76%	2556	6.36%	5780	3.68%
	IT	238	0.20%	208	0.52%	446	0.28%

### Implementation of automated DL algorithm

For the identification and classification of 156,965 radiographic images, a customized automatic DL engine (Neuro-T version 3.0.1, Neurocle Inc., Seoul, Korea) was adopted in this study. Within the available computing resources and training time, an automated DL algorithm is a self-training architecture that selects appropriate DL models and optimizes the hyperparameters (including the resize method, number of network convolutional layers, decay method, learning rate, dropout rate, batch size, number of epochs and patience, and optimizer) to fit the model in the customized dataset^36^.

The dataset comprised three groups: panoramic (*n* = 116,756), periapical (*n* = 40,209), and panoramic and periapical (*n* = 156,965) images. Each dataset group was randomly and evenly divided into three subgroups: training (80%), validation (10%), and testing (10%). After the dataset division, the training dataset was augmented by ten times, with random rotations (90°), hue (range of − 0.1 to 0.1), brightness (range of − 0.12 to 0.12), saturation (range of 0.6–1.5), contrast (range of 0.6–1.5), noise (0.05), and horizontal and vertical flips. We trained our approach on two NVIDIA A6000 graphic processing units (48 GB memory, NVIDIA, Mountain View, CA, USA). The models were trained for a maximum of 500 epochs and stopped if the validation set loss did not improve for more than 20 epochs.

### Statistical analysis

Categorical and continuous variables were expressed as frequencies (*n*) and ratios (%). The performance metrics were evaluated as accuracy, precision, recall, and F1 score (Eqs. (1)–(4), TP: true positive, FP: false positive, FN: false negative, and TN: true negative):

1 $$ {\text{Accuracy}} = \frac{{\left( {{\text{TP}} + {\text{TN}}} \right)}}{{\left( {{\text{TP}} + {\text{FP}} + {\text{TN}} + {\text{FN}}} \right)}} $$

2 $$ {\text{Precison }} = \frac{{{\text{TP}}}}{{\left( {{\text{TP}} + {\text{FP}}} \right)}} $$

3 $$ {\text{Recall}} = \frac{{{\text{TP}}}}{{\left( {{\text{TP}} + {\text{FN}}} \right)}} $$

4 $$ {\text{F}}1{\text{ score}} = 2{ } \times { }\frac{{\left( {{\text{Precision }} \times {\text{ Recall}}} \right)}}{{\left( {{\text{Precision }} + {\text{Recall}}} \right)}} $$

Additionally, a normalized confusion matrix for each DIS was calculated based on the test dataset. All data processing and statistical analyses were conducted using a commercial statistical package (Neuro-T version 3.0.1, Neurocle Inc., Seoul, Korea) and non-commercial statistical package (R version 4.2.0, R Foundation for Statistical Computing, Vienna, Austria).

## Supplementary Information

Supplementary Information.

## Data Availability

The dataset used in this study is a public dataset with limited access that can be used after approval by the National Information Society Agency (NIA), and details can be found on the AI-Hub website (https://aihub.or.kr/aihubdata/data/view.do?currMenu=115&topMenu=100&aihubDataSe=realm&dataSetSn=536).
