# Author Correction: Automated deep learning for classification of dental implant radiographs using a large multi-center dataset

**DOI:** 10.1038/s41598-023-33768-x

**Published:** 2023-04-21

**Authors:** Wonse Park, Jong-Ki Huh, Jae-Hong Lee

**Affiliations:** 1 Korean Academy of Oral and Maxillofacial Implantology (KAOMI) Implant Research Institute, Seoul, Korea; 2 Department of Advanced General Dentistry, Yonsei University College of Dentistry, Seoul, Korea; 3 Department of Oral and Maxillofacial Surgery, Gangnam Severance Hospital, Yonsei University College of Dentistry, 211 Eonju-ro, Gangnam-gu, Seoul, 06273 Korea; 4 Department of Periodontology, College of Dentistry and Institute of Oral Bioscience, Jeonbuk National University, 567 Baekje-daero, Deokjin-gu, Jeonju, 54896 Korea

Correction to: *Scientific Reports* 10.1038/s41598-023-32118-1, published online 24 March 2023

The original version of this Article contained an error in the spelling of the author Wonse Park which was incorrectly given as Won-Se Park.

The original Article has been corrected.

