# Supplementary material for: Automated deep learning for classification of dental implant radiographs using a large multi-center dataset
Source: Sci Rep. 2023 Mar 24;13:4862. doi: 10.1038/s41598-023-32118-1 (PMC10039053; doi:10.1038/s41598-023-32118-1)

APPENDIX 1. Consortium for dental implant dataset

All included protocols and datasets were supervised and managed by the National Information Society Agency (NIA) under the Ministry of Science and ICT and Korean Academy of Oral and Maxillofacial Implantology (KAOMI). Dental radiographic images were collected from five college dental hospitals and 10 private dental clinics, and the requirement for written informed consent was waived for the use of pre-existing and de-identified dataset. This dataset posted online at the AI-Hub website (https://www.aihub.or.kr)

1. Ethics

The large-scale and multicenter dataset used in current study was approved by the following Institutional Review Board (IRB): Seoul National University Dental Hospital (ERI21024), Yonsei University Dental Hospital (2-2021-0049), Gangnam Severance Dental Hospital (3-2021-0175), Wonkwang University Daejeon Dental Hospital (W2104/003-002), Dankook University Dental Hospital (2021-8-004), and national public IRB (P01-202109-21-020).

2. Consortium composition

The consortium composition and roles are as follows:

- Host organization (BC&Company Inc.): Project planning, general affairs, coordination of each participating institution, and legal/institutional research.
- Participating organization (KAOMI): Dataset collection, and primary verification of the dataset.
    - Five college dental hospitals: Seoul National University Dental Hospital (Department of Periodontology), Yonsei University Dental Hospital (Department of Advanced General Dentistry), Yonsei University Gangnam Severance Dental Hospital (Department of Oral and Maxillofacial Surgery), Wonkwang University Daejeon Dental Hospital (Department of Periodontology), and Dankook University Dental Hospital (Department of Periodontology).
    - Ten private dental clinics: S-plant Dental hospital, E-Well Dental Clinic, Boston Smart Dental

Clinic, Dr. Cho's Dental Implant Clinic, Win Dental Clinic, Yonsei Goun-miso Dental Clinic, Yonsei Haedam Dental Clinic, Seoul Top Dental Hospital, BOA Dental Clinic, and Yonsei Samsung Dental Clinic.

  - Dataset verification agency: Not involved in the current study

- Participating organization 2 (AiT-Story Corp.): AI solution development and quality control.
- Participating organization 3 (Belltechsoft): Data-building program development and quality control.

APPENDIX 2. NIA dataset

Table 1. Number of panoramic and periapical radiographic images for each dental implant system

Manufactures	System	Periapical images (n = 40,501)	Panoramic images (n = 125,199)	Total images (n = 165,700)
Biohorizon (n = 18)	Biohorizon external	10 (0.01%)	8 (0.00%)	18 (0.01%)
Cybermed (n = 444)	Core1	31 (0.02%)	413 (0.25%)	444 (0.27%)
Neobiotech (n = 21,260)	IS I	1,139 (0.69%)	6,708 (4.05%)	7,847 (4.74%)
	IS II	104 (0.06%)	2,774 (1.67%)	2,878 (1.74%)
	IS III	533 (0.32%)	7,594 (4.58%)	8,127 (4.90%)
	EB	518 (0.31%)	1,890 (1.14%)	2,408 (1.45%)
Nobel biocare (n = 5,542)	Branemark	342 (0.21%)	3,302 (1.99%)	3,644 (2.20%)
	Replace select	5 (0.00%)	1,893 (1.14%)	1,898 (1.15%)
Dentsply (n = 15,328)	Astra	571 (0.34%)	13,404 (8.09%)	13,975 (8.43%)
	Xive	654 (0.39%)	667 (0.40%)	1,321 (0.80%)
	Ankylos	10 (0.01%)	22 (0.01%)	32 (0.02%)
Dentis (n = 109)	S clean tapered	0 (0.0%)	109 (0.07%)	109 (0.07%)
Dentium (n = 41,139)	Implantium	4,162 (2.51%)	14,993 (9.05%)	19,155 (11.56%)
	Superline	5,207 (3.14%)	16,734 (10.10%)	21,941 (13.24%)
	Simpleline	13 (0.01%)	30 (0.02%)	43 (0.03%)
Dioimplant (n = 1,530)	UF	273 (0.16%)	525 (0.32%)	798 (0.48%)
	UF II	285 (0.17%)	447 (0.27%)	732 (0.44%)
Megagenimplant (n = 8,327)	Any ridge	135 (0.08%)	217 (0.13%)	352 (0.21%)
	Anyone internal	2,167 (1.31%)	1,640 (0.99%)	3,807 (2.30%)
	Anyone external	1,263 (0.76%)	1,290 (0.78%)	2,553 (1.54%)
	Exfeel external	115 (0.07%)	974 (0.59%)	1,089 (0.66%)
	Exfeel internal	71 (0.04%)	455 (0.27%)	526 (0.32%)
Biomet (n = 221)	3i	19 (0.01%)	202 (0.12%)	221 (0.13%)
Straumann (n = 4,977)	TS standard	176 (0.11%)	1,151 (0.69%)	1,327 (0.80%)
	TS standard plus	301 (0.18%)	741 (0.45%)	1,042 (0.63%)
	Bone level	1,261 (0.76%)	1,347 (0.81%)	2,608 (1.57%)
Shinhung (n = 3,393)	Luna	441 (0.27%)	2,935 (1.77%)	3,376 (2.04%)
	Stella	4 (0.00%)	13 (0.01%)-	17 (0.01%)
Osstem (n = 48,041)	GS II	327 (0.20%)	1,401 (0.85%)	1,728 (1.04%)
	GS III	43 (0.03%)	4,922 (2.97%)	4,965 (3.00%)
	SS II	116 (0.07%)	717 (0.43%)	833 (0.50%)
	TS III	10,536 (6.36%)	19,222 (11.60%)	29,758 (17.96%)
	US II	2,363 (1.43%)	7,295 (4.40%)	9,658 (5.83%)
	SS III	47 (0.03%)	109 (0.07%)	156 (0.09%)
	US III	185 (0.11%)	758 (0.46%)	943 (0.57%)

Warentec<br>(*n* = 15,102)	Hexplant	4,268 (2.58%)	4,565 (2.76%)	8,833 (5.33%)
	Internal	2,555 (1.54%)	3,216 (1.94%)	5,771 (3.18%)
	IT	208 (0.13%)	238 (0.14%)	446 (0.27%)
	IU	27 (0.02%)	25 (0.02%)	52 (0.03%)
Zimmer<br>(*n* = 199)	Screw vent	0 (0.0%)	12 (0.01%)	12 (0.01%)
	TSV	16 (0.01%)	171 (0.10%)	187 (0.11%)
Cowellmedi<br>(*n* = 70)	Atlas internal	0 (0.0%)	70 (0.04%)	70 (0.04%)

APPENDIX 3. Confusion matrix

Figures show the confusion matrix, with normalization, showing the results of the multiclass classification of dental implant systems.

Figure 1. Confusion matrix with normalization for classification of panoramic radiographic images

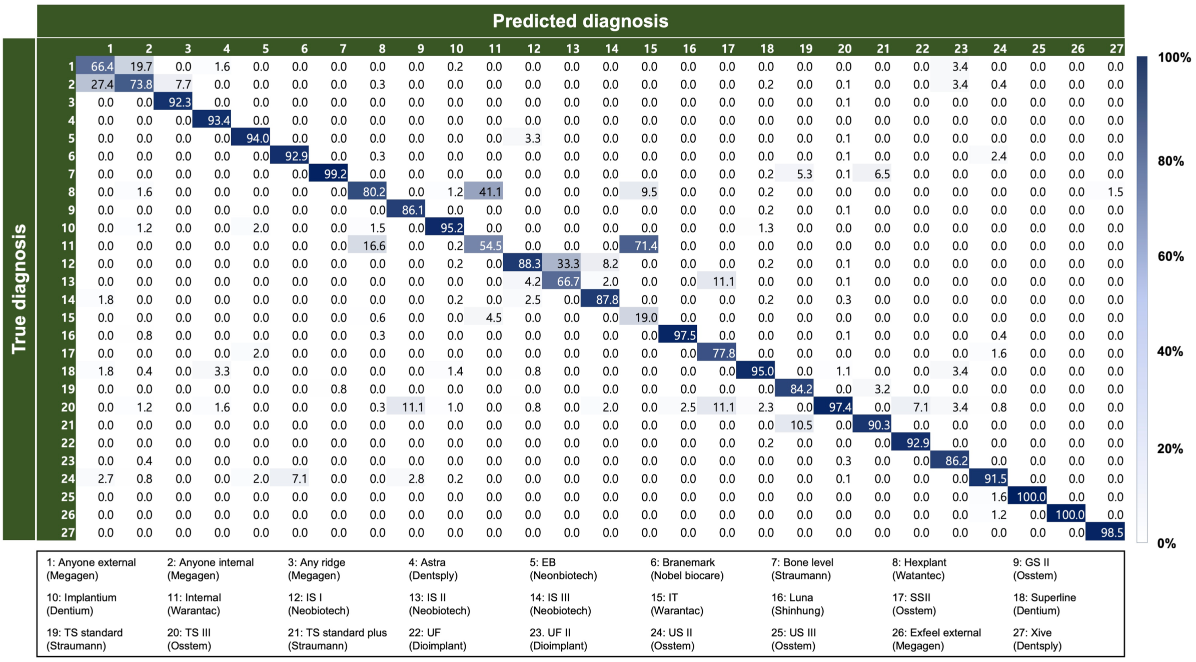

Figure 2. Confusion matrix with normalization for classification of periapical radiographic images

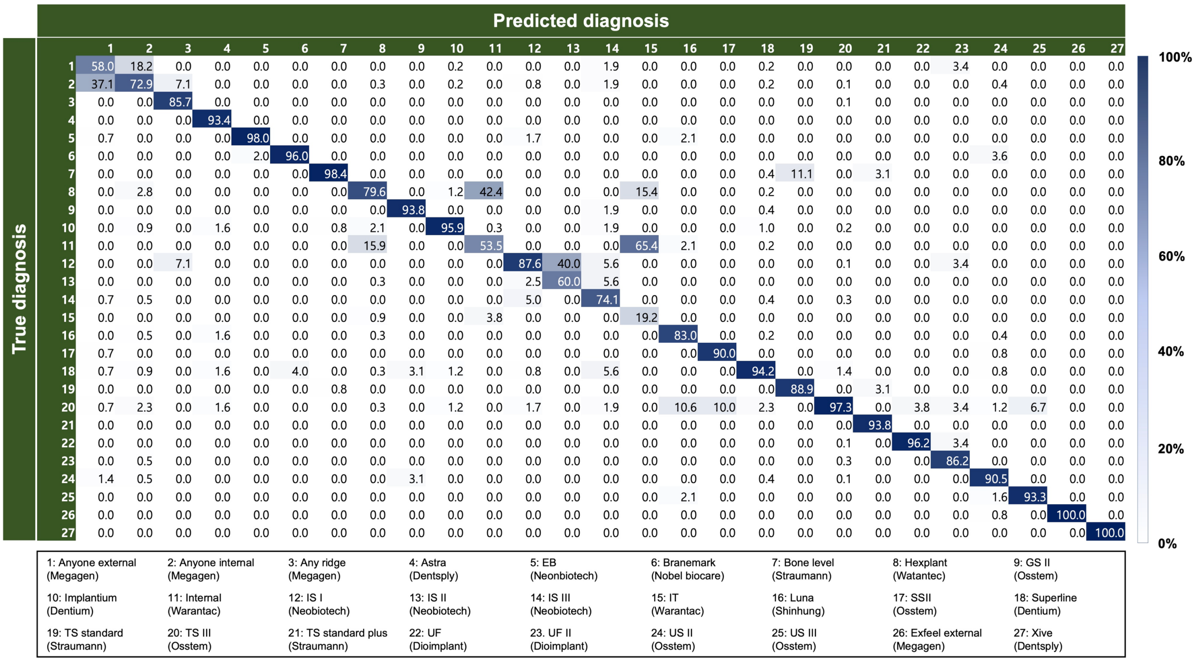

Supplement: Supplementary file 1 — Supplementary Information. [file 41598_2023_32118_MOESM1_ESM.pdf]
